# Decreasing packed red blood cell (pRBC) transfusions in neonates through quality improvement

**DOI:** 10.1038/s41372-026-02699-6

**Published:** 2026-04-23

**Authors:** Valerie Elberson, Kavya Rao, Sheetal Chepuri, Praveen Chandrasekharan

**Affiliations:** 1 Jacobs School of Medicine, Department of Pediatrics, SUNY at Buffalo, Buffalo, NY USA; 2 Division of Neonatology, SUNY at Buffalo, Buffalo, NY USA

**Keywords:** Paediatrics, Risk factors

## Abstract

**Objective:**

Using a quality improvement (QI) framework, we aimed to reduce the number of transfusions of packed red blood cells (pRBC) through adoption of evidence based more restrictive transfusion thresholds.

**Methods:**

A SMART aim was developed to decrease pRBC transfusions in infants less than 35 weeks gestation at birth by approximately 25 percent from the baseline rate of 28/1000 patient days by 12/31/2024 through implementation of a transfusion protocol with more restrictive thresholds. Five sequential Plan-Do-Study-Act cycles incorporated guidelines to decrease transfusions. Statistical Process Control charts (QI Macros SPC Software for Microsoft Excel) were used to track time-ordered data.

**Results:**

pRBC transfusions decreased from 28/1000 patient days to 18.3/1000 patient days below the target of 21/1000 patient days over a 16-month period.

**Conclusion:**

A transition to more restrictive transfusion thresholds was done successfully in our unit with a robust framework leading to fewer pRBC transfusions.

## Introduction

### Problem description

Preterm infants because of their clinical condition and inherent physiology often receive transfusion of blood products [1–3]. Research has focused on the potential negative outcomes related to transfusion of adult blood products to preterm neonates. Studies have shown associations of increased mortality and morbidities related to prematurity such as bronchopulmonary dysplasia (BPD), necrotizing enterocolitis (NEC), retinopathy or prematurity (ROP) and neurodevelopmental impairment (NDI) with increasing number of packed red blood cell (pRBC) transfusions [4–9]. It is hypothesized that the adult blood products are pro-inflammatory, and may trigger inflammation or dysregulated immune responses in preterm neonates [10–12]. The biophysical properties of fetal/neonatal hemoglobin are very different from adult hemoglobin [13]. Replacing fetal hemoglobin with adult hemoglobin in neonates has been shown to be associated with increased risk for BPD, ROP and intraventricular hemorrhage (IVH) [14, 15]. For these reasons benefits vs. the potential for harm needs to be assessed when making decisions related to transfusions in preterm neonates.

### Available knowledge

In 2006 the Premature Infants in Need of Transfusion (PINT) study showed that in extremely low birth weight (ELBW) infants using a higher threshold for transfusions resulted in more infants receiving transfusions, but failed to show evidence of improved outcomes [16]. More recently, large multi-center randomized control trials (RCTs) have supported the use of more restrictive transfusion thresholds [17, 18]. The Transfusion of Prematurity (TOP) trial concluded that, a higher hemoglobin threshold for pRBC transfusion in ELBW infants did not improve survival without NDI at 22 to 26 months of corrected gestational age [17]. The Effects of Liberal vs. Restrictive Transfusion Thresholds (ETTNO) trial, similarly, concluded that a liberal blood transfusion strategy did not reduce the likelihood of death or disability at 24 months compared with a restrictive strategy in ELBW infants [18]. Consensus recommendations have been put out to support more restrictive pRBC transfusion practices [19, 20]. Centers have reported safely introducing more restrictive pRBC thresholds [21, 22].

### Rationale

As a level IV regional perinatal center, we recognized the need to decrease thresholds to avoid unnecessary transfusions. We planned a quality improvement (QI) initiative to transition to more restrictive transfusion thresholds for pRBCs.

### Specific aims

A SMART (specific, measurable, achievable, relevant, time-bound) aim was developed with the following goal: to decrease pRBC transfusions in infants less than 35 weeks by 25 percent from the baseline rate by 12/31/2024. The SQUIRE (Standards for Quality Improvement Reporting Excellence) 2.0 guidelines were used for writing this publication [23].

## Methods

### Context

The Children’s Hospital of Buffalo neonatal intensive care unit (NICU) is a 64-bed level IV regional neonatal perinatal center with approximately eight to nine hundred admissions per year located in Buffalo, New York. We are both a birthing facility and a referral center for outborn admissions. Once an infant has been discharged from the hospital, they are not typically readmitted to our NICU except in rare circumstances. Participants included providers in the NICU who order blood transfusions (ie, neonatal nurse practitioners, neonatal physician assistants, pediatric residents, neonatal fellows, and attending neonatologists).

### Interventions

**Fig. 1:**
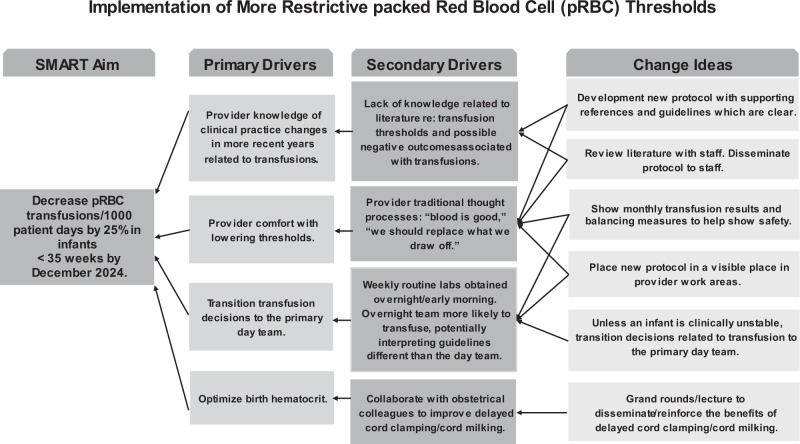
Key Driver Diagram. Linking the SMART aim to actionable drivers and change ideas for implementation of more restrictive packed red blood cell (pRBC) thresholds.

In December of 2023, we conducted a literature review and gathered baseline data. A key driver diagram was developed with the SMART AIM specific to the target population, key drivers and change ideas (Fig. 1). Baseline data was gathered through a query of the electronic medical record (EMR). In January 2024 our initial PDSA (Plan-Do-Study-Act) cycle incorporated an education session with discussion of key articles, along with presentation of the new protocol and baseline data. The protocol was disseminated to providers in February 2024 as our secondary PDSA cycle. This was done through email updates and short presentations. We also did staff education related to results from the large RCT transfusion studies TOP and ETTNO [17, 18]. To help with the transition, another PDSA cycle made the protocol accessible in the EMR and a hard copy of the protocol was posted near provider workstations. The protocol was developed with a transfusion strategy based on the criteria used in the TOP trial (Supplement 1) [17]. Despite the thresholds and clinical criteria being significantly different than the old protocol, our center had been a part of the TOP trial so the new thresholds were familiar to providers.

It is known that inadequate iron supplementation can predispose the preterm neonate to suboptimal erythropoiesis [24, 25]. We decided to put an additional focus on our clinical management related to anemia. Iron optimization was reviewed with the rounding teams weekly with nutrition and pharmacy input. We also engaged our obstetrics colleagues to optimize our birth hematocrit with a focus on delayed cord clamping (DCC) and umbilical cord milking (UCM) when appropriate through a grand rounds presentation for another PDSA cycle [26]. While assessing our key drivers, one of the biggest concerns we had identified was related to pRBC transfusion decision making. Ordering for transfusions was occurring in the overnight shift as routine labs were drawn in the early hours of the morning. We prioritized having the primary/day team make transfusion decisions, given their greater familiarity with the patient and adherence to guidelines.

### Ethics approval

This QI initiative was registered with the University at Buffalo and was approved for exemption based on its intended purpose of quality improvement within the institution.

### Measures and analysis

Data were gathered from a local database (Neodata) and extracted from the EMR (Cerner Powerchart) for the total number of pRBC transfusions to assess the preintervention baseline to plan guideline changes and prospective data collection. To normalize data, patient days were calculated as the number of patients in the NICU for each day of the month selected.

A baseline rate of pRBC transfusions was calculated by collecting data on all pRBC transfusions in infants less than 35 weeks for 2022 and 2023 per 1000 patient days. Our aim was to reduce pRBC transfusions in infants less than 35 weeks by 25 percent from baseline of 28 transfusions per 1000 patient days. For a process measure, protocol compliance was tracked monthly. It was planned to evaluate approximately 25 percent of the monthly transfusions for protocol compliance. This was designated as 8 randomly picked transfusions per month. Total monthly pRBC transfusions were also tracked as a process measure. Balancing measures included mortality, rate of necrotizing enterocolitis (NEC using modified Bell staging criteria), intraventricular hemorrhage (IVH), oxygen dependance at 36 weeks corrected gestational age and retinopathy of prematurity (ROP)

Statistical Process Control (SPC) charts were used to assess time ordered data and assess for special cause variation. The Institute for Healthcare Improvement (IHI) rules for control charts were utilized. QI Macros SPC Software for Microsoft Excel was used for chart development [27]. Statistical analysis of baseline characteristics and balancing measures were done by calculating a *p* value from z scores for 2 population proportions.

## Results

**Fig. 2:**
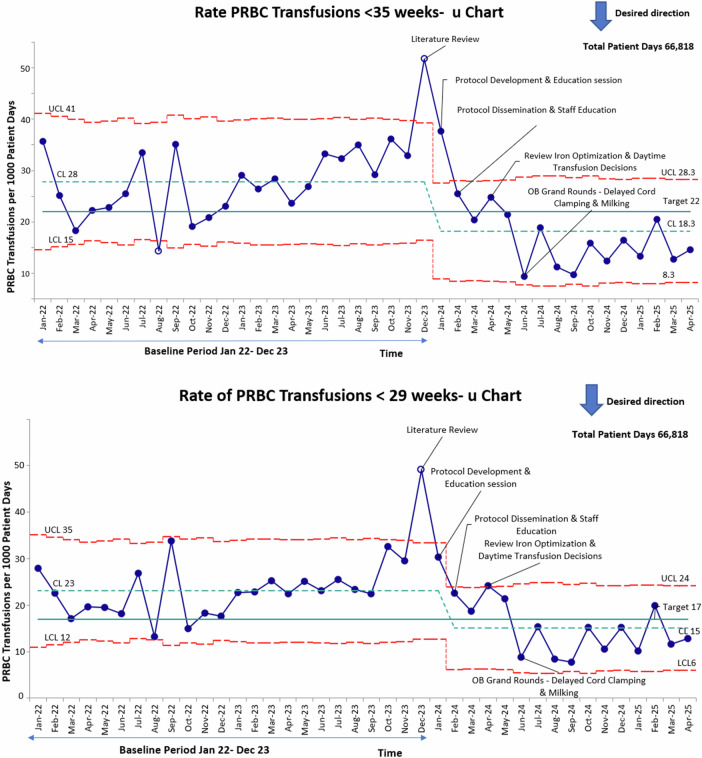
Laney U Chart-displaying monthly pRBC transfusions per 1000 patient days in infants born between 22 and 35 weeks gestational age from January 2022 through April 2025. In both less than 35 weeks gestation and in less than 29 weeks gestation, pRBC transfusions per 1000 patient days trended downward below the target line after project implementation. Interventions are annotated. Center line (mean) was adjusted after special cause variation was noted. Red lines indicate the upper control limit and lower control limit. UCL-Upper control limit. LCL-Lower control limit. CL-Center line.

For the primary outcome measure the target was 21 transfusions per 1000 patient days from a baseline of 28 transfusions per 1000 patient days. This target was achieved reducing our pRBC transfusions to 18.3 per 1000 patient days (Fig. 2). We further subdivided our population into infants less than 29 weeks with the same target of approximately 25 percent reduction from a baseline of 23 per 1000 patient days to a target of 17 per 1000 patient days. The target was surpassed down to 15 per 1000 patient days (Fig. 2).

During the baseline period, PRBC transfusion rates demonstrated common cause variation, fluctuating around the initial centerline (CL = 28 transfusions per 1000 patient days) and remaining within control limits, indicating a stable but higher transfusion rate. Following the implementation of the QI interventions in early 2024 including development and dissemination of a standardized transfusion protocol, provider and nursing education, optimization of iron supplementation, and obstetric grand rounds focused on delayed cord clamping and cord milking which showed a distinct and sustained downward shift in PRBC transfusion rates that was observed beginning in January 2024. Eight or more consecutive data points fell below the prior centerline, meeting SPC criteria for special cause variation and signifying a true process improvement.

A new centerline was established at 18.3 transfusions per 1,000 patient days, representing an approximately 35 percent reduction from baseline. The process subsequently stabilized, showing common cause variation around the new centerline and maintaining transfusion rates consistently below the target threshold of 21 per 1000 patient days. These findings demonstrate a statistically and clinically significant reduction in PRBC transfusions sustained over more than 12 months following implementation of standardized transfusion practices and multidisciplinary education.

Similarly, we were able to achieve more than a 25 percent reduction in even the smallest patients, confirmed by dividing out the less than 29 week population. Their baseline was 23 per 1000 patient days with a target of 17 per 1000 which was achieved below that at 15 per 1000 patient days. This was also an approximately 35 percent decrease from baseline.

**Fig. 3:**
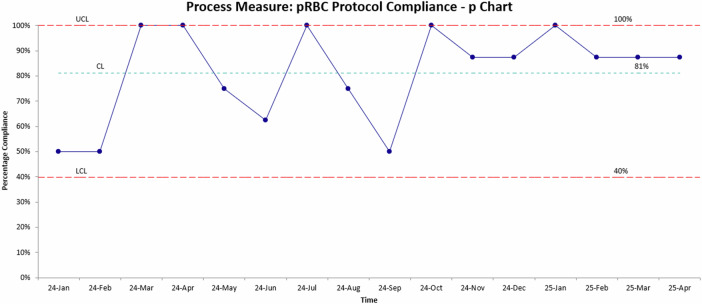
p-Chart for process measure demonstrating pRBC transfusion protocol compliance. This data was based on ~8 random chart audits per month. Implementation of the standardized transfusion protocol is indicated, followed by sustained adherence in transfusion practices across subsequent months.

**Fig. 4:**
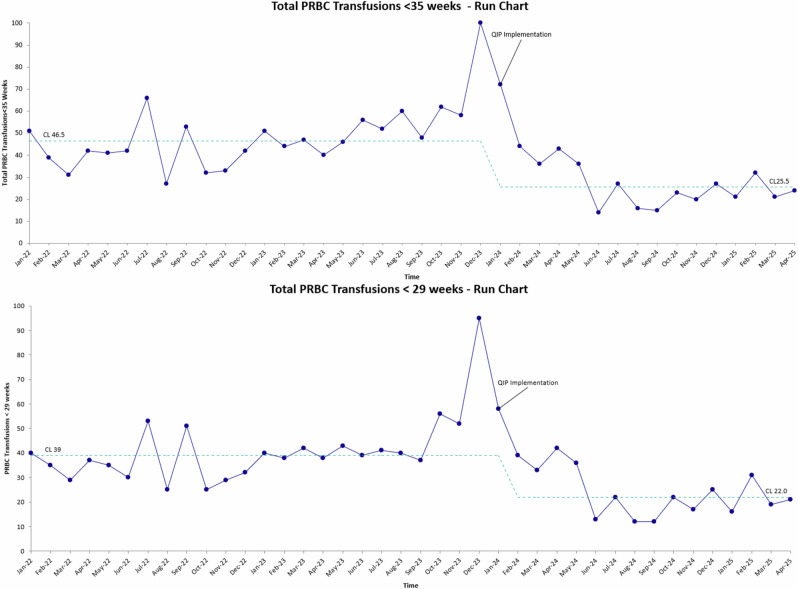
Run chart illustrating decreased pRBC transfusion frequency following initiation of the quality improvement intervention. The post-implementation period shows a consistent decline compared with baseline. CL-Center line.

Each month, approximately 8 transfusions (25% of the monthly average number of transfusions) were randomly assessed for protocol compliance and opportunities for education as a process measure. Overall compliance was 81% (Fig. 3). Non-compliant transfusions were typically within 0.5 g/dL of the threshold number. Total monthly transfusions were also tracked (Fig. 4). The less than 35 week centerline dropped from 46.5 down to 25.5. Likewise, the less than 29 week centerline dropped from 39 down to 22.

Baseline characteristics and balancing measures including NEC, IVH, death, oxygen dependence at 36 weeks corrected gestation and ROP were similar between the two time periods (See Table 1).

**Table 1:** Baseline Characteristics/Balancing Measures.

Total Admissions	1720	1043	
**Males**	956 (55.6%)	577 (55.3%)	0.90
**Infants** < **35 Weeks**	783 (45.5%)	450 (43.1%)	0.22
**Infants** < **29 Weeks**	150 (8.7%)	91 (8.7%)	1
**NEC Stage 2 or Higher**	29 (1.7%)	22 (2.1%)	0.42
**IVH Grade 3 or 4**	25 (1.5%)	11 (1.1%)	0.37
**Death**	25 (1.5%)	14 (1.3%)	0.81
**VLBW O2 @ 36 Weeks**	103/258 (39.9%)	57/145 (39.3%)	0.90
**Any ROP**	86 (5%)	56 (5.4%)	0.67

*p* value calculated by using *z* score for 2 population proportions.

## Discussion

Anemia of prematurity, specifically in the very low birth weight (VLBW) and extremely low birth weight (ELBW) infant is a major concern for those who take care of neonates. Physiologic anemia in the smallest patients is exaggerated by the need for blood draws, decreased iron stores and low levels of erythropoietin [28, 29]. It is recognized that many infants in the NICU will receive transfusions with reports as high as 40% in the VLBW and 90% in the ELBW population [1, 29]. Potential associations of morbidities with increasing number of pRBC transfusions, and the report of harm from the Planet-2/Matisse platelet trial in the higher threshold group, the risk/benefit must be weighed when deciding who should be transfused [4–9, 30, 31]. Several recent studies have shown that lower pRBC thresholds can be used without an increase in mortality or NDI [16–18]. Based on this information we decided to move forward with an updated evidence-based protocol for pRBC transfusion for our level IV NICU.

Through our quality improvement initiative, we intended to change thought processes regarding pRBC transfusions at all levels including physicians, advanced practice providers and nurses. There were several key factors that we felt helped with our success. Disseminating the research that supported the new protocols including large multi-center RCTs helped convince providers related to safety concerns [17, 18]. We also emphasized the increasing evidence for potential worse outcomes related to increased number of transfusions [4–9]. Making the new protocol readily available since it was a significant change from the previously used protocols and transitioning decision making related to transfusions to the primary daytime clinical teams for stable patients allowed for greater adherence to the protocols. With other recent reports of successfully reducing thresholds for platelet transfusions, we also decided to lower thresholds for platelet transfusions at the same time to put an overall emphasis on less blood product use/transfusions in the NICU [32–34].

### Limitations

There are several limitations to this quality initiative. This is a relatively short time frame. We recognize this as an important clinical success, but understand the need to make sure that this is a sustained change. We also understand that we have more opportunity to further decrease our transfusion rate with other interventions. We can infer compliance based on our improvement in transfusion rates and the random compliance audits that we did monthly, but also acknowledge that transfusions of pRBCs can be difficult to evaluate for protocol compliance as there is ambiguity related to determining the need for transfusion secondary to clinical condition. Some of the cases which were deemed non-compliant included infants with spells, poor growth or concerns of symptomatic anemia such as persistent tachycardia. Going forward this could be a potential focus area to determine if there are specific issues that need to be addressed related to compliance to further decrease transfusion rates. We assume cost savings secondary to our overall decreased usage of blood products, but we did not gather institutional data to support this.

### Future directions

To support long-term sustainability, we will continue to emphasize adherence to the established protocol. With the transition to a new EMR in the coming year, we anticipate additional opportunities to further reduce pRBC transfusions by implementing complementary interventions. These include standardizing laboratory testing particularly during the first week of life for VLBW infants such as leveraging cord blood for initial labs when feasible, linking transfusion protocols directly to transfusion orders, and exploring the development of an order set or protocol for erythropoiesis-stimulating agents in selected populations. We understand that this project is just an initial step towards continued decreased transfusions in our unit in a relatively short time frame, but we feel it is a very clinically important success for our unit and patients.

## Conclusion

Implementation of a standardized, evidence-based transfusion protocol coupled with multidisciplinary education resulted in a measurable and sustained improvement in our NICU. The SPC analysis demonstrated clear episodes of special cause variation following intervention, confirming true process changes rather than random fluctuation. This shift reflects enhanced adherence to transfusion thresholds, improved team awareness, and stronger alignment with current neonatal transfusion guidelines. The subsequent stability and persistence of improvement over more than a year indicate that the interventions not only reduced unnecessary transfusions but also established a reliable and sustainable process. From a clinical perspective, the reduction of pRBC transfusions supports safer transfusion stewardship, minimizing donor exposure and potential transfusion-related complications in preterm and critically ill neonates. The success of this initiative underscores the effectiveness of multidisciplinary collaboration, standardized education, and continuous data monitoring as key strategies in driving durable improvements in NICU transfusion practices.

## Supplementary information

Old pRBC Transfusion Protocol Compared with New pRBC Transfusion Protocol

## Data Availability

The datasets generated during and/or analyzed during the current study are available from the corresponding author on reasonable request.
