# Supplementary material for: Decreasing packed red blood cell (pRBC) transfusions in neonates through quality improvement
Source: J Perinatol. 2026 Apr 23;46(5):868–73. doi: 10.1038/s41372-026-02699-6 (PMC13190257; doi:10.1038/s41372-026-02699-6)
Supplement: Supplementary file 1 — Old pRBC Transfusion Protocol Compared with New pRBC Transfusion Protocol [file 41372_2026_2699_MOESM1_ESM.docx]

**New Protocol**

**Transfusion Thresholds pRBCs**

**Respiratory Support No Respiratory Support**

**Period: Hgb(g/dL)/Hct Hgb(g/dL)/Hct**

**Week 1** 11/32 10/29

**Week 2** 10/29 8.5/25

***Week 3*** 8.5/25 7/21

****Respiratory support-CPAP, noninvasive ventilation, or ventilation and FiO2 more than 0.35. Transfusion at a lower FiO2 may be considered based on the clinical condition of the patient.**

****HFNC or NC with an FiO2 more than 0.35 will be a team clinical discussion based on the individual patient.**

Typical pRBC volume 15ml/kg with an infusion time of approximately 2h.

*A lower volume or shorter/longer time of infusion may be considered depending on patient condition.

*In clinical situations which could be concerning for symptomatic anemia, transfusion may be considered at a lower threshold. Examples might include sepsis, tachycardia, or recurrent apnea/bradycardia spells.

*For clinically stable infants, transfusion in the first 72h of life should be based on a clinical team discussion.

*Decisions related to transfusion based on routine lab monitoring will generally be made during daytime clinical rounds.

Kirplani et al. Eunice Kennedy Shriver NICHD Neonatal Research Network. Higher or Lower Hemoglobin Transfusion Thresholds for Preterm Infants. N Engl J Med 2020; 383:2639-2651.

**Old Protocol**

**PRBC TRANSFUSION GUIDELINES**

**Hct < 20%** (Give 2 x 10 ml/kg PRBC's over 2 hr. each)

• Signs or symptoms of anemia

• Tachycardia, tachypnea, poor feeding, OR

• Low reticulocyte count ( < 4‐6%)

**Hct < 30%** (Give 15 ml/kg PRBC's over 2 hr)

• On < 35% hood O2

• On O2 by nasal cannula

• On CPAP and/or ventilator with MAP < 6cm H2O

• Recurrent apnea or bradycardia on therapeutic level of Rx

o > 6 episodes in 12 hr, OR

o 2 episodes requiring BVM in 24 hr.

• Significant tachycardia

o > 180 beats/min for 24 hr.

• Significant tachypnea

o > 80 breaths/min for 24 hr.

• Poor weight gain on adequate calories

o < 10 GM/day over 4 days

**Hct < 35%** (Give 15 ml/kg PRBC's over 2 hr) • on > 35% hood O2

• On CPAP and/or ventilator with MAP greater than or equal to 6cm H2O

**Hct < 40%** (Give 10 ‐ 15 ml/kg PRBC's over 1/2 ‐ 1 hr) • if acute blood loss

o Tachycardic

o Hypotensive

• With Congenital Heart Disease

• On ECMO

Clinics in Perinatology 2000;27(3):734‐735

Transfusion 2002;42:1398‐1413
